# Intumescent Polymer Metal Laminates for Fire Protection

**DOI:** 10.3390/polym10090995

**Published:** 2018-09-06

**Authors:** Laura Geoffroy, Fabienne Samyn, Maude Jimenez, Serge Bourbigot

**Affiliations:** Univ.Lille, ENSCL, UMR 8207, UMET, Unité Matériaux et Transformations, F 59 000 Lille, France; laura.geoffroy@univ-lille.fr (L.G.); fabienne.samyn@ensc-lille.fr (F.S); maude.jimenez@univ-lille.fr (M.J.)

**Keywords:** polymer metal laminates (PML), fire protection, intumescent paint

## Abstract

Intumescent paints are applied on materials to protect them against fire, but the development of novel chemistries has reached some limits. Recently, the concept of “Polymer Metal Laminates,” consisting of alternating thin aluminum foils and thin epoxy resin layers has been proven efficient against fire, due to the delamination between layers during burning. In this paper, both concepts were considered to design “Intumescent Polymer Metal Laminates” (IPML), i.e., successive thin layers of aluminum foils and intumescent coatings. Three different intumescent coatings were selected to prepare ten-plies IPML glued onto steel substrates. The IPMLs were characterized using optical microscopy, and their efficiency towards fire was evaluated using a burn-through test. Thermal profiles obtained were compared to those obtained for a monolayer of intumescent paint. For two of three coatings, the use of IPML revealed a clear improvement at the beginning of the test, with the slopes of the curves being dramatically decreased. Characterizations (expansion measurements, microscopic analyses, in situ temperature, and thermal measurements) were carried out on the different samples. It is suggested that the polymer metal laminates (PML) design, delays the carbonization of the residue. This work highlighted that design is as important as the chemistry of the formulation, to obtain an effective fire barrier.

## 1. Introduction

Nowadays, in transportation and building applications, fire safety is crucial, and materials must meet stringent regulations. Fireproofing solutions have consequently been developed to comply with more demanding standards. Among them, intumescent coatings (IC) are known as an efficient and widely used solution. When these coatings are submitted to a thermal constraint, they swell and form a porous insulating barrier, so-called “char”, protecting the underlying substrate from heat and mass transfers. Most intumescent coatings contain three main ingredients: An acid source, a carbon source, and a blowing agent [1,2,3,4,5,6,7]. The formulation of the coating must be optimized in terms of physical and chemical properties, to form an effective protective char. Even though novel chemistries and additives are under investigation to increase coatings efficiency and to reduce thickness and weight, novel designs of intumescent coatings have reached a plateau. The tendency now is to play with fibers or meshes to improve thermal protective properties and mechanical resistance of the char.

**Figure 1:**
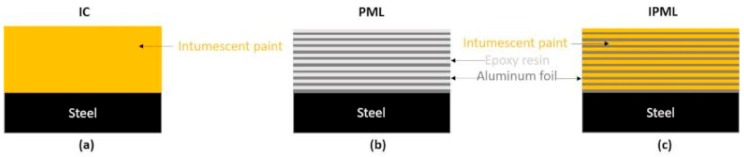
Illustration of (**a**) intumescent coatings (IC), (**b**) polymer metal laminates (PML), and (**c**) intumescent polymer metal laminates (IPML) systems.

Another approach, especially in the aeronautics field, consists of using fiber metal laminates, such as ARALL (alternating thin aluminum alloy layers and uniaxial or biaxial aramid fiber prepreg) and GLARE (similar to ARALL, but with glass fibers instead of aramid fibers [8,9,10,11]). Based on this concept, Polymer Metal Laminates (PML) were developed. PML are a hybrid composite structure (Figure 1b), of alternating thin aluminum foils and thin epoxy resin layers glued on substrates, such as carbon epoxy composite or aluminum plates. This design, combining the advantages of metallic and polymeric materials, shows great mechanical and thermal performance [12,13]. Moreover, one advantage of PML is that it contains many interfaces susceptible to delaminate, which can improve the fireproof property of the substrate.

The main idea in this paper was to set up an original concept, by gathering both technologies previously reported (i.e., IC, Figure 1a and PML, Figure 1b), to design novel and highly efficient protective thermal barriers, i.e., Intumescent Polymer Metal Laminates (IPML, Figure 1c).

Ten-plies IPMLs were designed, alternating thin aluminum foils and intumescent coatings, and glued onto steel substrates using a thin epoxy resin layer. Three different coatings of various chemistries (i.e., epoxy based, acrylic based, and silicon based) were evaluated. The IPMLs designed, were characterized by optical microscopy and tested against fire using a bench-scale test (mimicking a burn-through). Fire protective performances of the IPMLs were compared to reference materials, i.e., the neat intumescent coatings applied on steel plates, of similar weight and thickness. To try to explain the different fire behaviors observed, expansion and in situ temperature measurements, as well as thermal and microscopic analyses, were carried out.

## 2. Materials and Methods

### 2.1. Materials

We used as substrate for all experiments, 100 × 100 × 3 mm^3^ steel (grade XC38) plates, purchased from Tartaix (French company specialized in metal and tooling, Paris, France). These plates were previously sandblasted (Normfinish, Jean Brel SA, Stains, France) using a white aluminum oxide (Guyson, Chambly, France) (particle size around 355–500 µm), at a pressure of 5 bars, and cleaned with acetone (VWR, Radnor, Pennsylvania, United States) before use. From Carl Roth (Karlsruhe, Germany), 30 µm thick Rotilabo aluminum foils of high purity (99%) were purchased. The three fireproofing coatings selected to prepare the PML, are reported in Table 1. They were all designed for cellulosic (ISO834) or hydrocarbon (UL1709) fire scenarios. A and B were conventional commercial epoxy and acrylic based coatings, respectively, containing classical intumescent agents and designed to pass UL 1709 and ISO 834, respectively. Coating C was a silicone-based coating designed by our team, as described in Reference [14], containing expandable graphite as the main additive. Physical expansion was thus observed under heating, due to expansion of the graphite. Mechanical cohesion was reported to be obtained through the formation of Si-C bonds [12,13,15,16,17].

### 2.2. IPML Elaboration Process

**Figure 2:**
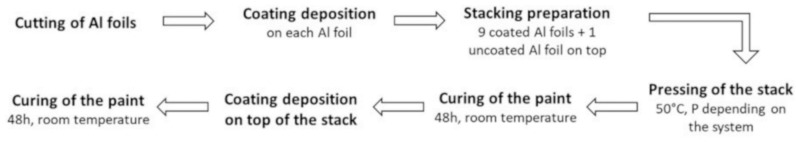
IPML elaboration process.

IPML processing occurred in several steps, illustrated in Figure 2. Aluminum foils were first cut into 100 mm × 100 mm squares. Then, intumescent coatings (respectively A, B, and C coatings) were manually deposited onto the aluminum foils. Three stacks of ten aluminum foils and nine intumescent coating layers were prepared and pressed using a press supplied by Fontijne Grotnes B.V. (Vlaardingen, Netherlands), to reduce the void content due to air entrapment. For coatings A and C, a force was applied, starting at 10 to 20 kN, with a ramp of 0.9 kN/min at 50 °C. Then, the load was set at 20 kN for 1 min. For paint B, due to the different chemistry and viscosity of the coating (acrylic based), a 10 kN load was applied for 12 min at 50 °C.

After that, a last thin layer of coating (A, B, and C, respectively equal, at 300, 160, and 170 µm (measured using an optical microscope)) was applied to the top of the aluminum foils to form the final IPML, which was then cured for 48 h at room temperature. Finally, it was glued onto a steel plate, using an epoxy resin (DGEBA, purchased from Sigma-Aldrich, St louis, MO, USA, reference 31185) cross-linked with Diethylenetriamine, (purchased from Sigma-Aldrich, St. Louis, MO, USA, reference D93856) with a ratio of 100:11.7. The whole system (plate + IPML) was cured again, for 48 h at room temperature.

Using this process, IPMLs made of 10 aluminum foils with layers of paints A, B, or C were prepared and glued onto steel plates. They were named IPML-A, IPML-B, and IPML-C, and their total thicknesses were 8, 5.5, and 5 mm, respectively (Table 2).

To provide an accurate comparison between IPML systems and monolayers of intumescent coatings, some additional samples were prepared, namely IC-A, IC-B, and IC-C. Steel plates were covered using a film applicator, with respectively, coating A (IC-A), coating B (IC-B), and coating C (IC-C), and their total thicknesses were 8, 5, and 4.8 mm, respectively (Table 2). Uncoated steel plate was also used as a reference for each fire test.

### 2.3. Fire Testing

#### 2.3.1. Burn-Through Test

**Figure 3:**
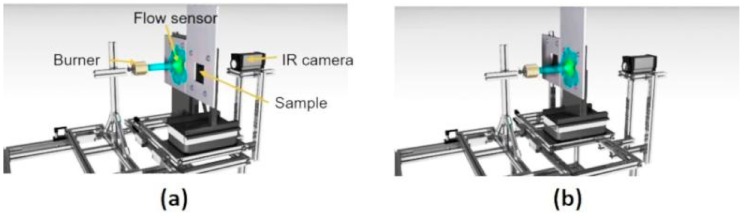
Burn-through test bench illustration [18] (**a**) Flame calibration, (**b**) Switch between calibration to sample.

The selected lab scale fire test (fully described elsewhere, in Reference [18]), consisted of exposing the samples to a 116 kW/m^2^ heat flux, using a propane torch, as illustrated in Figure 3. This test bench was compliant with two aeronautical certification fire tests: ISO2685:1998(E) and FAR25.856(b):2003.

At the beginning of the test, each sample was maintained between two 10 mm thick insulating panels from FINAL Advanced Materials (Calsil) and attached using four screws. The test was carried out without any ventilation, to avoid the influence of convection. Before exposing the samples to fire, a calibration of the propane flame was done for 5 min to check that the heat flux was constant and equal to 116 kW/m^2^. Then, the samples were exposed to the flame for 15 min and cooled at room temperature.

During the test, temperature versus time profiles were measured at the backside of the samples, using a K-type thermocouple (TC SA, Dardilly, France) welded onto the middle of the backside of the steel plates. Data were recorded using an Agilent 34970A data logger (Keysight Technologies, Santa Rosa, CA, USA). The temperature evolution of samples was also measured using an infrared camera (FLIR thermovision A40, Wilsonville, OR, USA), placed at a fixed distance to the backside of the steel plate. All sample backsides were previously spray-coated with a heat resistant mat black paint (Jelt noir mat 700 °C supplied by ITWPC Spraytec, Asnières-sur-Seine, France), ensuring that they all had the same surface emissivity (close to 0.9). All the IR measurements were recorded using Thermacam research professional 2.0. software (FLIR system, Wilsonville, OR, USA), and computed using Research IR software (FLIR system, Wilsonville, OR, USA). Each experiment was repeated at least 3 times, to check the repeatability of the fire test results.

#### 2.3.2. Thermal Gradient Measurement

**Figure 4:**
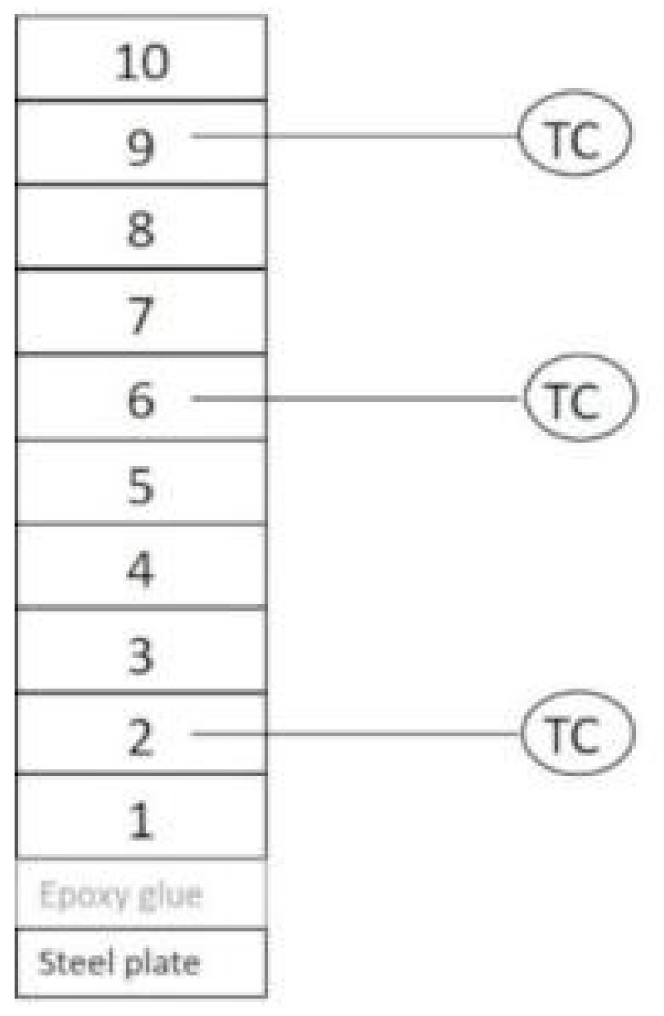
Illustration of thermal gradient measurement in IPML.

To better understand the fire protective behavior of some IPML systems, the thermal gradient inside these systems was measured: Three thermocouples were inserted between aluminum foils (before press step), as shown in Figure 4. Then the PML was pressed, cured, and coated, as previously described. Finally, IPML containing thermocouples were exposed to fire for 15 min.

### 2.4. Characterizations

#### 2.4.1. Microscopic Analyses

Before fire testing, optical microscopy observations were carried out on 1 × 1 cm^2^ IPML cross sections using a microscope VHX-1000 HDR (High Dynamic Range), Keyence (Osaka, Japan). Preparation of the cross sections was as follows: Each sample was put in liquid nitrogen for 5 min, and then cut using a blade and a hammer. Using this protocol, a brittle fracture was obtained without affecting the integrity of the sample. Samples obtained were then embedded in an epoxy resin, dried for 48h at room temperature, and polished (up to 1/4 μm) using silicon carbide disks (ESCIL, Chassieu, France) to obtain the smoothest surface as possible to facilitate observations.

Scanning Electron Microscopy (SEM) observations were performed on residues obtained after fire testing, using a Hitachi S4700 (Tokyo, Japan). The residues were cut and fixed on the SEM holder using carbon conductive double-face adhesive tape, supplied by Nisshin EM Co., Ltd. (Tokyo, Japan). Then, samples were carbon coated using a Bal-Tec SCD005 sputter coater (BAL-TEC, Pläffikon, Switzerland).

#### 2.4.2. Thermal Analyses

Thermogravimetric analyses (TGA) were carried out on IPML residues obtained after fire testing, using a TG 209 F1 Libra supplied by Netzsch (Selb, Germany). When possible, residues were collected between each aluminum foils. They were then ground in a mortar and for each experiment; 10 mg samples were positioned in aluminum open pans, on a gold sheet to avoid possible reactions of the phosphorus species of the coating with the alumina pans.

The thermograms were recorded in the 40–800 °C temperature range, with a heating rate of 10 °C/min under nitrogen flow, Air Liquide grade (40 mL·min^−1^).

## 3. Results

### 3.1. IPML Characterizations before Fire Test

**Figure 5:**
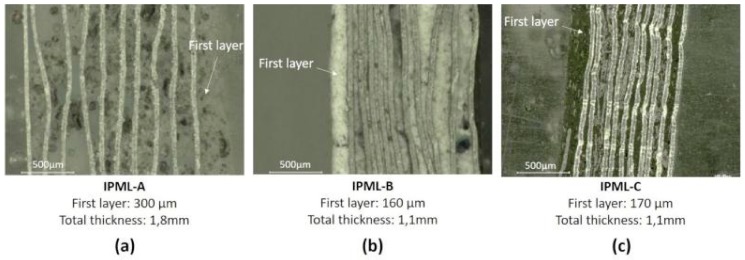
Cross section pictures obtained by microscopic analyses of (**a**) IPML A, (**b**) IPML-B, and (**c**) IPML-C, before burn-through test.

A cross-section of each IPML (respectively named IPML-A, IPML-B, and IPML-C) before testing was observed using an optical microscope, and the resulting pictures are gathered in Figure 5. All IPMLs seemed quite homogeneous, with no voids observed between aluminum foils. Moreover, the IPMLs have almost the same thickness of intumescent paint between each aluminum foil. The mean thickness gap between each aluminum foil was calculated for each IPML and corresponded to 70, 105, and 77 µm, for IPML-A, B, and C, respectively. The thickness of the upper coating layer (applied after press step) was different and depended on the coating applied: 300 µm for IPML-A, and 160, and 170 µm, for IPML-B and IPML-C, respectively. It was observed that intumescent layers in IPML-B and C were thinner compared to IPML-A. This was explained by the difference in terms of viscosity between the coatings: The coating A was very thick and difficult to apply because it contained many fillers and fibers, preventing the obtaining of very smooth layers. However, even if thicknesses of the IPML-A, B, and C were different, they can be considered as acceptable as (i) results were repeatable (repeatability measurements were made on three different samples), and (ii) the IPMLs will not be compared to each other in the rest of the study, but would be compared to single intumescent layers of similar thicknesses, i.e., IC-A, IC-B, and IC-C.

### 3.2. Fire Performances

**Figure 6:**
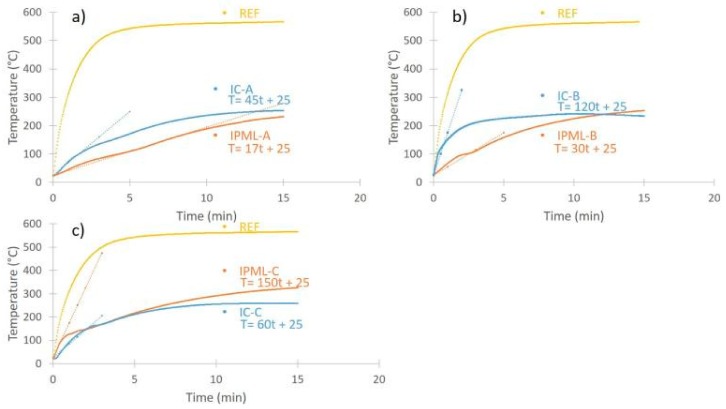
Evolution of temperature versus time for each system ((**a**) Temperature vs. time for A system, (**b**) Temperature vs. time for B system, (**c**) Temperature vs. time for C system).

Fire protection performances of IPML-A, B, and C were compared to those of IC-A, B, and C of almost similar total thickness (around 8, 5.5, and 5 mm, respectively). Figure 6 and Table 3 report the temperature versus time curves and values measured at the backside of the uncoated (REF) and coated steel plates, for the different systems.

First, it was observed that all coated systems, whatever IC or IPML, were very efficient in protecting steel against fire: The backside of the reference plate reached about 550 °C in a few minutes, whereas IC and IPML systems allowed limiting backside temperature increase to maximum 300 °C.

**Figure 7:**
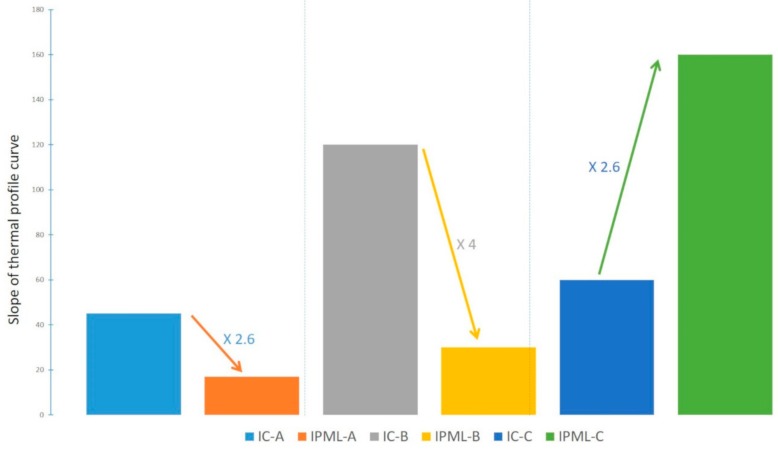
Slope comparison of thermal profile curve at the beginning of burn-through test.

The IPML-A design (Figure 6a, Table 3) is more advantageous than a monolayer of intumescent coating A. Indeed, during the first minutes of the test, the slope of the curve of IPML-A was 2.6 times lower than that of the IC-A system (Figure 7), leading to a maximum temperature difference of about 65 °C after 5 min 50 s. In addition, at the end of the test, the temperature reached by IPML-A was still slightly lower (22 °C difference), than that measured for IC-A. In that case, the IPML design clearly allowed improving of the fire protection performance of the system. For the IPML-B system (Figure 6b, Table 3), similar observations were done, but at the beginning of the test the benefit of using IPML was even more obvious than for the previous system. The slope of the curve was strongly reduced (factor 4) (Figure 7), and a maximum temperature difference between both systems of about 102 °C after 2 min 50 s was observed. At the end of the test, the temperature reached by IPML-B was 19 °C higher than the temperature reached by the IC-B system, which was not significant (incertitude range of the measurements).

Therefore, both IPML-A and IPML-B systems provide a real benefit in terms of fire protection, compared to single coating layers (IC-A and IC-B). It is of interest to manage to reduce the temperature at the beginning of the test. This is usually managed, for a short time, by using additives dehydrating during the first minutes of the test, but here, through the IPML design, the effect lasted for more than 10 min. 

IPML-C system (Figure 6c, Table 3), on the contrary, does not exhibit any improvement compared to the IC-C system. The thermal protection was even better without IPML design. Indeed, at the beginning of the fire test, the slope of the IPML-C system increased by 2.6 compared to the IC-C system (Figure 7). At the end of the fire test, the temperature reached by the IPML-C system was also higher than the one reached by IC-C (difference of 70 °C after 15 min, Table 3). Based on this result, the IPML design seemed not to be working for coating C system.

**Figure 8:**
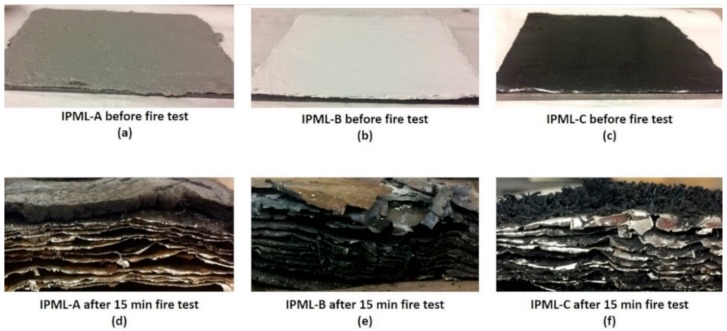
Cross-section of IPML after 15 min burn-through test exposition ((**a**) IPML-A before fire test, (**b**) IPML-B before fire test, (**c**) IPML-C before fire test, (**d**) IPML-A after 15 min fire test, (**e**) IPML-B after 15 min fire test, and (**f**) IPML-C after 15 min fire test).

To try to understand and explain the results obtained, cross-sectional pictures of IPML residues obtained after the 15 min burn-through test, are presented in Figure 8. In cases A and B, swelling and delamination were observed, due to some carbonaceous and cohesive residue formation between aluminum foils. These observations confirmed all expectations triggered by the PML design itself (delamination phenomenon). These phenomena might explain the good fire protection properties of the A and B systems, previously reported.

However, the case of the C coating was different. In that system, some swelling was also observed, as well as delamination. However, as reported previously, coating C is a silicon-based system containing expandable graphite, and its efficiency is mainly based on a “physical expansion”: The entangled network between the “worms” of graphite creates an efficient heat barrier, leading to its good fire protective properties. However, due to the design of the IPML, the intumescent layers were not in direct contact: the thin swelled layers containing expandable graphite were not thick enough to lead to a formation of an entangled network. “Worms” of expandable graphite grow perpendicularly to aluminum foils, which form thermal bridges between aluminum and graphite. Thus, IPML-C was not able to yield a comparable protection, compared to the single 5 mm thick IC-C layer (which formed an entangled network with holes reducing thermal propagation), explaining the lower fire protective properties.

As IPML-C exhibited poor performance compared to the two other systems, the rest of the paper will focus on IPML-A and B, to try to understand why and how both these systems work.

It is well known that expansion plays a crucial role in the efficiency of intumescent coatings, especially if it is combined with the right char porosity and morphology [18]. Therefore, expansion and morphological analyses were carried out and are described in the following sections.

### 3.3. Expansion Measurements

**Figure 9:**
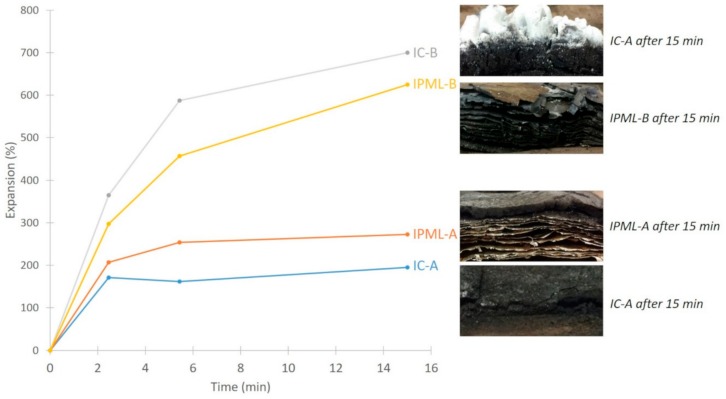
Expansion versus time curves for IC-A, IC-B, IPML-A, and IPML-B systems.

The objective here was first to evaluate expansion during the fire test, by stopping the test at characteristic times, depending on the thermal profiles obtained in the previous section. Three characteristic times were selected: 2 min 50 s, 5 min 50 s, and 15 min (end of the test). Based on the thermal profiles, at 2 min 50 s the gap between the IC-B and the IPML-B was the highest (102 °C), and at 5 min 50 s the gap between IC-A and IPML-A was the highest (65 °C). Expansions were measured after 2 min 50 s, 5 min 50 s, and 15 min, and the expansion versus time curves obtained are plotted in Figure 9. These curves revealed that, after 2 min of testing, the expansion of IPML-A was higher than that of IC-A, whereas for the other system, from the beginning of the test, the expansion of IPML-B was lower than that of IC-B. IC-A and IPML-A reach in a few minutes a “plateau” corresponding to about 180% and 250% expansion, respectively. Whereas IC-B and IPML-B still expanded after the 15 min test, and expanded much more than the A system, with final expansions between 600 and 700%. No clear conclusion could be drawn from these measurements, as both systems were efficient but clearly worked in a different manner.

Based on these experiments, it is well known that expansion must be correlated to a morphological analysis [19] to confirm the good fire barrier phenomenon, because it is at least as important as the expansion study. Thus, a morphological analysis was undertaken to try to clarify the results obtained.

### 3.4. Char Morphology Analysis

**Figure A1:**
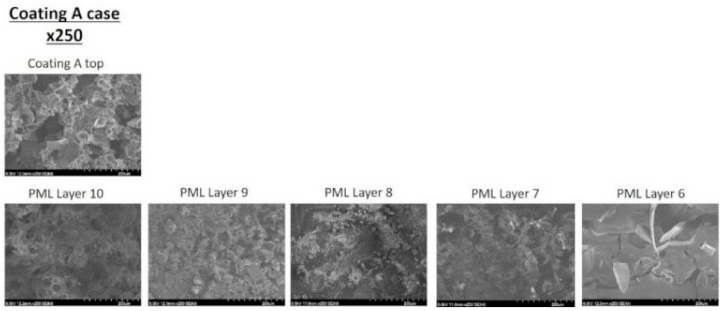
SEM pictures comparing IC-A char to IPML-A chars (taken from layers 10 to 6).

**Figure A2:**
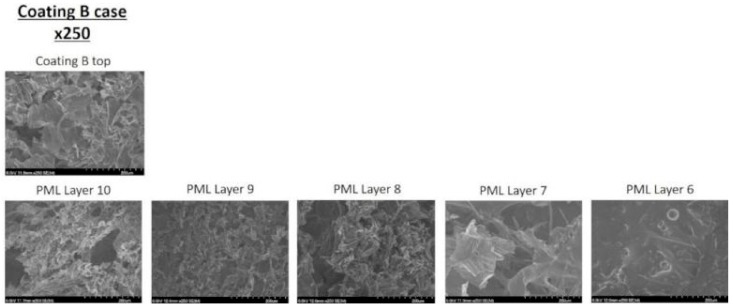
SEM pictures comparing IC-B char to IPML-B chars (taken from layers 10 to 6).

The IPML-A and IPML-B chars obtained at the end of the fire test were analyzed by SEM and compared to IC-A and IC-B final chars, respectively (Figure A1 and Figure A2 in Appendix A). For IPML systems, chars taken from the layer 10 (surface) to layer 6 were analyzed.

Unfortunately, for both systems, due to the very thin layers of intumescent coating between aluminum foils, leading to a low amount of carbonaceous residue, observations were difficult. Looking at the SEM pictures, it was rather hard to conclude on a modification of the char structure. Sole interesting observation was that from layer 6 to layer 1, in both IPML systems, no porosity was observed, as if no intumescent char had developed, whereas on the contrary, the upper layers were where porosity could be observed. This might probably mean that there was a high thermal and carbonization gradient inside the IPML, which could explain the good fire protective effect observed. Thus, based on these observations, there are some differences, which will be studied in the next section.

### 3.5. Thermal and Carbonization Gradients

**Figure 10:**
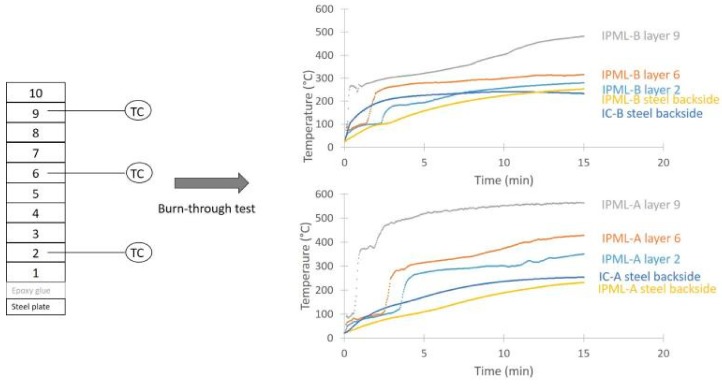
Thermal gradient evolution in IPML-A and IPML-B.

A thermal gradient was measured in both IPML-A and IPML-B systems, following the set-up described in the experimental section. Figure 10 highlights the evolution of the temperature versus time in the layers 2, 6, and 9, in which thermocouples were incorporated. The curves obtained clearly showed a thermal gradient in both cases. For IPML-A, after a 15 min fire test, the temperature reached 232, 350, 428, and 562 °C, for the steel backside, and for the layers 2, 6, and 9, respectively. For IPML-B, the temperature reached 253, 279, 316, and 482 °C, for the steel backside, and the layers 2, 6, and 9, respectively. The thermal gradient between the backside of the plate and layer 9 of the IPML, was about 330 °C for IPML-A (initial thickness 8 mm) and 229 °C for IPML-B (initial thickness 5.5 mm).

**Figure 11:**
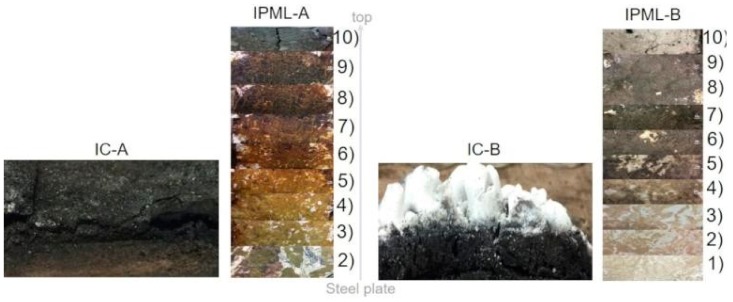
Carbonization gradient evolution in IPML-A and B, compared to IC-A and IC-B chars (Layer 10 is the top layer (directly exposed to fire) for IPML-A and IPML-B).

**Figure 12:**
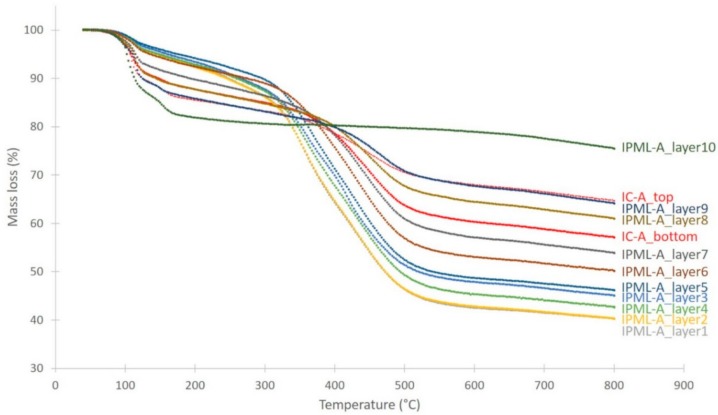
TG curves for each char layer of IPML-A and for IC-A char layer, obtained after 15 min burn-through test exposure.

**Figure 13:**
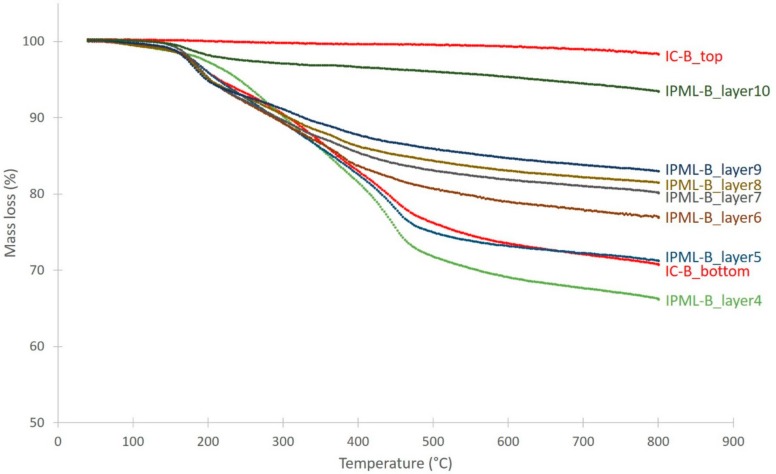
TG curves for each char layer of IPML-B and for IC-B char layer, obtained after 15 min burn-through test exposure.

Related to this thermal gradient, a carbonization gradient is also observed (Figure 11). The color of each residual layer appears different, involving different carbonization degrees: The top layer exposed to fire is much darker, and thus more carbonized than the last layer. In addition, this carbonization gradient was much clearer with the IPML design than with IC systems. Moreover, using the thermal gradient measurement, the carbonization gradient was highlighted. The layer 9 reached a temperature above 560 and 480 °C, for IPML-A and IPML-B, respectively, which confirmed intumescent coating degradation, and the visual aspect of the residue. For layer 2, the temperature was under 350 and 280 °C, for IPML-A and IPML-B, respectively. Using TGA measurement in Figure 12 and Figure 13, the thermal degradation of the coating occurs at around 400 and 300 °C, for IC-A and IC-B, respectively. Therefore, thermal degradation had just started in this layer after 15 min fire exposition. These observations confirmed better fire protection performance. 

To obtain quantitative information on this carbonization gradient, char retrieved from each layer of IPML-A and IPML-B was submitted to thermogravimetric analyses (TGA) to estimate its residual organic degree at the end of the fire test, compared to the TGAs of the top and bottom parts of the IC-A and IC-B systems (Figure 12 and Figure 13, Table 4). Therefore, most residues were further analyzed by TGA. It is important to notice that no TGA measurements were done for residues of layers 1 and 2 of IPML B, because aluminum foils were too strongly glued, and no residue could be retrieved.

For both IPML systems, the carbonization gradient was obvious. IPML-A showed a gradient ranging from 25% organic content (top-layer 10) to 63% (bottom-layer 1), corresponding to a 38% difference between the top and bottom layers. For the IC-A char, a difference of only 8% of organic content was reached between the top and bottom layers. It was also observed that from layer 7, the coating was less degraded than in the IC system. Similar observations were done for the IPML-B system. IPML-B showed a gradient from 7% (top-layer 10) to 34% (layer 4), corresponding to a difference of 27%. The same difference (27%) is registered between the top and bottom layers of the IC-B char. These differences were quite similar, but one must not forget that it was not possible to analyze layers 1, 2, and 3, which were obviously less degraded than the other ones.

**Figure 14:**
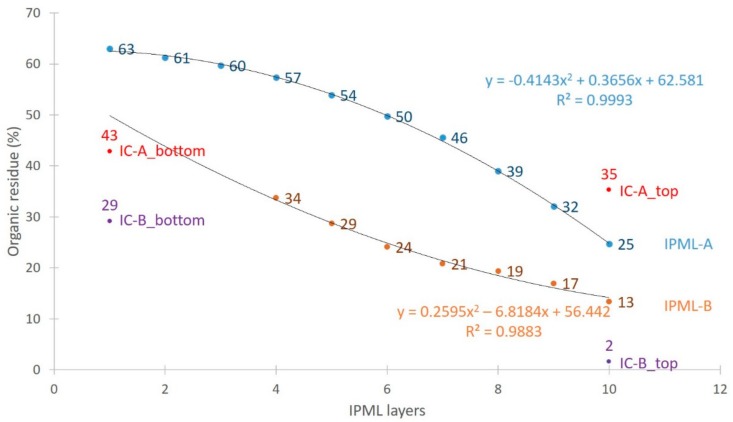
Evolution of carbonization gradient depending on the IPML layer number.

The evolution of the organic degree for IPML-A and IPML-B was plotted versus the IPML layer numbers (Figure 14), and curves were fitted with polynomial equations (*y* = −0.4143*x*^2^ + 0.3656*x* + 62.581, *R*² = 0.9993 for IPMLA; and *y* = 0.2548*x*^2^ − 6.748*x* + 56.194, *R*² = 0.9883 for IPML-B. This fitting was done to estimate the organic degree in layer 1, 2, and 3 in IPML-B. Moreover, the organic degree of IC-A top and bottom, and IC-B top and bottom, is indicated in Figure 14. For both systems with coatings A and B, it is clear that the organic degree is higher for the IPML design than for the IC.

## 4. Discussion

To conclude on these results, a new concept was evaluated, namely Intumescent Polymer Metal Laminates, which was compared to neat intumescent coatings to estimate the PML design advantage. Three intumescent coatings based on three different chemistries (epoxy, acrylic, and silicone) were tested. In the case of a physical expansion (silicon-based resin containing expandable graphite), IPML design did not improve the fire protection of the steel substrate. On the contrary, the steel backside reached higher temperatures quicker. As explained previously, no entangled network is formed between expandable graphite “worms” and aluminum foils due to too thin layers applied in-between the aluminum foils, and the higher constraint that multi-layer design triggers. In the IPML-C system, “worms” of graphite were oriented perpendicularly to aluminum foils. Owing to this “worms” orientation, some thermal bridges were created between aluminum foils and “worms” of graphite, increasing the heat propagation. On the opposite, when IC-C was exposed to fire, physical “worms” expansion occurred, and an entangled network (through graphite entanglement) with holes was formed. As a result, an efficient heat barrier, leading to good fire protective properties, was ensured. IC-A and IC-B are classical intumescent coatings relying on the chemical intumescent process based on degradation of the carbon source, dehydration of the acid source, and release of gases from the swelling agents. For these systems, using the PML design allowed improved thermal protection properties, particularly during the first ten minutes of the test. Thermal analyses and instrumentation of the IPML with thermocouples, showed that high temperature and carbonization gradients are obtained, much higher than those obtained in the sole IC chars. Differences in terms of expansion and char morphologies, not being obvious between the IPML and IC samples, the explanation of this fire protection improvement could be (i) the delamination effect observed between layers, and (ii) the chemical intumescent phenomenon. It created a heat gradient, and hence a large carbonization gradient. Therefore, when the IPML design is exposed to fire, the first intumescent layer starts to swell and decompose. This phenomenon protects other layers from heat propagation for a certain time. Then, the heat slowly spreads through the aluminum foil to the second intumescent layer. After a certain temperature is reached, the second intumescent layer starts to decompose and swell. This swelling creates a delamination in between aluminum foils. In addition to the intumescent phenomenon, it improved the fire protection performance of the IPML system, because of air heat insulation properties, arising from delamination. Afterwards, the heat is spread to other intumescent layers, and the same mechanism occurs in each layer. Thus, it is the combination of intumescence and delamination, that the material can reach better thermal protection properties. The thermal gradient between layers is consequently more important, leading to a higher carbonization gradient.

**Figure 15:**
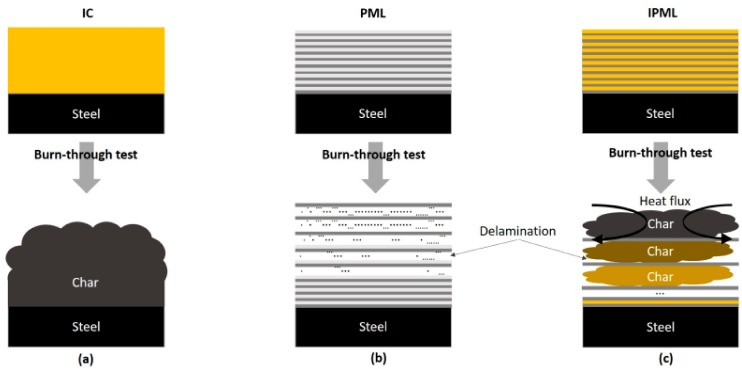
Illustration of the thermal behavior when ((**a**) IC, (**b**) PML, and (**c**) IPML) are exposed to a high heat flux fire test.

Therefore, Figure 15 summarizes the different mechanisms of action of the IC, the PML, and IPML. In the IC case, only intumescence occurs to protect the steel substrate from the heat. With PML design, fire protection is brought by delamination (epoxy resin decomposes and produces gas). With the new IPML design, as it was previously explained, both phenomena were combined (chemical intumescence and delamination), which led to good heat protection performance.

## 5. Conclusions

A new design was elaborated and tested in this paper: PML systems combined with intumescent paints, namely IPML. Three kinds of fireproof paints were studied, exhibiting different mechanisms of action. It was evidenced that using an intumescent coating relying on physical intumescence (due to expandable graphite), the IPML system is of no advantage compared to a sole intumescent coating layer. Indeed, fire protective properties are not improved in that case, which can be explained by the fact that aluminum foils probably disturb the physical expansion of the coating. However, the IPML concept shows strong interest, compared to a monolayer of paint, when intumescent coatings relying on chemical intumescence processes are used. These IPML systems clearly improve fire protection, particularly during the first ten minutes of the fire tests. This is explained by the combination of the chemical intumescent process and delamination observed in between the aluminum foils. Both these phenomena increased the thermal and carbonization gradient in the IPML system, and thus improved heat protection performance on the steel substrate.

Currently development of novel intumescent coatings is reaching a limit due to the limit in “novel chemistries”. Thanks to these first series of experiments, IPML design was proven as an efficient concept and a unique opportunity to improve fire protection without changing the formulation chemistry.

## Figures and Tables

**Table 1:** Intumescent coatings characteristics

Paint	Targeted Fire Scenario	Type of Paint	Type of Intumescence
A	UL 1709	Epoxy, bi-component [3,4,5,6,7]	Chemical
B	ISO 834	Acrylic, mono-component	Chemical
C	UL 1709, ISO 834	Silicone, bi-component [12,13,15,16,17]	Physical

**Table 2:** IC and IPML characterization.

Samples	Thickness (mm)	Weight (g)
Steel + IC-A	8.0	288
Steel + IPML-A	8.0	291
Steel + IC-B	5.0	257
Steel + IPML-B	5.5	249
Steel + IC-C	4.8	272
Steel + IPML-C	5.0	269

**Table 3:** Temperature reached after 2, 10, and 15 min burning, for each sample studied.

Samples	Thickness (mm)	T Reached after 2 min Burning (°C)	T Reached after 10 min Burning (°C)	T Reached after 15 min Burning (°C)
REF	3.0	437	561	567
IC-A	8.0	110	236	254
IPML-A	8.0	67	189	232
IC-B	5.5	191	241	234
IPML-B	5.0	96	225	253
IC-C	5.0	147	255	256
IPM-C	4.8	147	291	326

**Table 4:** Organic residue of IPML-A and IPML-B for each layer.

IPML Layers	Organic Residue of IPML-A (%)	Organic Residue of IPML-B (%)
1	63	Cannot be measured
2	61	Cannot be measured
3	60	Cannot be measured
4	57	34
5	54	29
6	50	23
7	46	20
8	39	19
9	32	17
10	25	7
IC-top	35	2
IC-bottom	43	29
